# Evaluating the effects of Escanbil (Calligonum) extract on the expression level of Catsper gene variants and sperm motility in aging male mice

**Published:** 2014-07

**Authors:** Mahsa Askari Jahromi, Mansoureh Movahedin, Zohreh Mazaheri, Masoud Amanlu, Seyed Javad Mowla, Hosein Batooli

**Affiliations:** 1 *Department of Anatomy, Faculty of Medical Sciences, Tarbiat Modares University, Tehran, Iran.*; 2 *Department of Medicinal Chemistry, Faculty of Pharmacy, Drug Design and Development Research Center, Tehran University of Medical Sciences, Tehran, Iran.*; 3 *Department of Genetic, Faculty of Basic Sciences, Tarbiat Modares University, Tehran, Iran.*; 4 *Isfahan Research Center of Agriculture and Natural Resources, Kashan Botanical Garden, Kashan, Iran.*

**Keywords:** *Spermatozoa*, *Catsper protein*, *Antioxidants*, *Ca*^*2+*^* channels*

## Abstract

**Background:** Catsper proteins are responsible for entering Ca^2+^ to the cell and play an important role in sperm motility and male fertility. Antioxidants are vital for sperm motility too. Escanbil (Calligonum) extract possess some of the important antioxidant like Catechin and Quercetin.

**Objective:** Here we investigated the effects of Escanbil (Calligonum) extract on the sperm parameters and the expressing of Catsper gene in aging male mice.

**Materials and Methods: **In this animal study, firstly, dose response was performed by using these three doses of Escanbil (Calligonum) (10, 30 and 50 mg/kg). 5 mice in each group were considered and Intra Peritoneal injection was done for 5 weeks. the sperm parameters analyzed and dUTP nick end labeling (TUNEL )staining was done. 30 mg/kg dose was considered as optimum dose. Secondly: fifteen aging male mice (11-13 months) were divided into three groups: control, sham and experiment. The experiments were injected Intra peritonealy with Escanbil (Calligonum) extract (30mg/kg) weekly for up to 5 weeks. The sham group was injected Intra Peritoneal (DMSO). Sperm parameters were analyzed. Expression of Catsper genes was analyzed by Real time PCR.

**Results: **Our results showed that after Escanbil (Calligonum) treatment (30 mg/kg), the sperm parameters were improved in experimental group (p<0.05). Our data showed that there was a statistical significance difference between the expressions of Catsper 2, 4 in aging experiment group comparison with aging control group (p<0.05).

**Conclusion:** We investigated that the Escanbil (Calligonum) extract (30 mg/kg) can improve sperm parameters and change the expression of Catsper genes in aging male mice. This herbal extract can be used as an antioxidant component for clinical usages.

## Introduction

Aging has an impact on male infertility. By aging, the sperm count, the sperm motility and the sperm normal sperm morphology decrease slightly (1). Despite the fact that some men can become fathers up to age 80 or older, couples should consider the effects of aging on both partners when making parenting plans and decisions. It was showed that oxidative damage in infertile men is decuple of fertile men (2). Weyrobek *et al* have reported that aging in male causes DNA fragmentations and results in Dwarfism. So With age, the chance of having a healthy baby decreases (3). The efficient function of sperm depends on factors such as, high motility, viability, normal morphology, and sufficient number (count). It is possible to improve sperm motility naturally through nutrient supplementation. Because decreased sperm motility is one of the signs of increased oxidative stress in the body (4). 

Male infertility due to low sperm motility is dependent on the loss of specific nutrients in the diet, poor life style choices, and especially oxidative stress. Low sperm motility and sperm count increase as a result of poor dietary habits. Some of the enzymes like: superoxide dismutase, glutathione peroxidase, glutathione reeducates, catalases can catalyze reactions to counteract reactive oxygen species (5). These enzymes are called endogenous defense system. These enzymes also require co-factors such as selenium, iron, copper, zinc and manganese for optimum catalytic activity (6). A sufficient dietary comprising these materials may cause the amplifying of these antioxidant defense mechanisms.

Some of these antioxidants exist in the fruits and vegetables (7). The *Escanbil (Calligonum) *Commosum is a plant which has the important antioxidants like Catechin, Epicatechi, Quercetin, kamefrol and Genistin (8). Catechin is a type of natural phenols and antioxidant which is a plant secondary metabolite (9). It is often considered to belong to the family of flavonoids. A study showed that Catechin, Quercetin and flavonoids functions require the interaction with glutathione-peroxidase enzyme (10). Scientists have reported that Catechin could improve the sperm motility in boar (9). Quercetin has inflammatory characteristies and can change the expression of TNFα in vitro. Using and absorption of these important minerals may decrease with aging.

Ca^2+^ is a vital element for sperm motility, capacitation and acrosomal reaction (11). One of the important calcium channels which has recognized in mammalian cells are Catsper channels (12). Catsper1 was discovered by Ren. Catsper1 codes a protein which has located in plasma membrane of sperm mid piece (13). Catsper1 protein is a ca^2+^ channel which plays an important role in sperm motility (14). Catsper2 is important for sperm motility too (15). Scientists have reported that Catsper 3, 4 are important in acrosomal reactions (16). Cutting off calcium channel resulted in losing the power of fertilization by sperm (17). Scientists are trying to find some ways to turn the calcium channel on, but we would also need to turn it on at the right time.

Reactive oxygen species (ROS) are molecules that are highly disruptive to cellular function. These radicals are produced by sperm for hyper activation, capacitation, acrosomal reaction, oocyte penetration, and signal transduction (18). The oxidative stress is a misbalance between the ROS generation and antioxidants in the cells. These radicals in sperm are responsible for as much as 40% of male infertility. The sperm cell is highly sensitive to damage by the reactive oxygen molecules because of its exclusive structure. The plasma membrane of sperm has a lot of unsaturated fatty acids which are highly sensitive to oxidative damage which ultimate decreasing of sperm motility, capacitation, and sperm binding to the egg zona pellucida (19).

So we tried to answer the following questions: firstly is Catsper gene expression affected by aging? Secondly: can *Escanbil (Calligonum) *treatment impress the expression of Catsper gene in aging male mice? In this study we investigated the effects of *Escanbil (Calligonum) *extract on Catsper gene expression. We analyzed the sperm parameters in aging and young male mice and concentrated on Catsper genes expression in these groups.

## Materials and methods

**Preparation of extract**

The ethanolic *Escanbil (Calligonum) *Comusum extract was prepared by faculty of pharmacy in Tehran University and dissolved in DMSO. Briefly, *Escanbil* fruit were washed thoroughly in water, air dried for a week at 35-40ºC and pulverized in electric grinder. The powdered samples were macerated in methanol (Merck, Germany) and petroleum benzene boiling range 40-60^o^C (Merck, Germany) for 72 hours and filtered by 0.45 mm pore size Millipore filters. The residue was washed twice with ethanol 96% and filtrate was evaporated to produce sticky dried material of *Escanbil* extract and stored at 4ºC until use. 

**Dose response in animal**

Based on an experimental study, we achieved optimum dose of *Escanbil (Calligonum) *extract in 2 stages. All procedures were approved by the Tarbiat Modares University Ethics Committee. At the first stage, we achieved dose response for three doses (10, 30 and 100 mg/kg) according to pharmacology standard method, one mouse in each group was considered and injected for 5 weeks, after this period the mice were sacrificed and sperm parameters were analyzed. 

According to these data, does 30 mg/kg of *Escanbil (Calligonum) *extract was determined as optimum dose. So the range of other doses determined according to this dose. At the second stage, we achieved dose response for three doses (10 mg/kg, 30 mg/kg, and 50 mg/kg). Five mice were considered in each group and sperm parameters were analyzed after 5 weeks again. TUNEL staining in these groups were achieved too.

**Evaluation of catsper gene expression**

Fifteen 11-13 months aging male mice and fifteen 2-3 months aging male mice weighing 25-30 g were purchased from Tarbiat Modares University. The animals were maintained under standard conditions. They got free foods and water. The mice were divided into three groups: control, sham and experiment. The experiment group was injected with *Escanbil (Calligonum) *extract (30 mg/kg; IP) weekly for up to 5 week. The sham group, was injected with DMSO (IP) for 5 weeks. After this period, animals were sacrificed by cervical dislocation and sperm parameters were analyzed. One of the testes from each mice was used for Real time PCR. We used GAPDH gene as housekeeping. Our data analyzed by spss using ANOVA test.

**Sperm analysis in the experimental groups**

For sperm analyze, epididymis of each mice were removed and placed in the PBS. The epididymis was minced in PBS and sperm cells were allowed to swim into PBS for 30 min at 36^o^C. One drop of the sperm suspension put on a slide for light microscope observation of motility. The motile and immotile sperm cells were numbered under the 400× magnification. In order to reduce the error, rate of 200 spermatozoa were counted in each sample. Neubaur haemocytometer slide was used for sperm count. For estimating the number of sperm with normal morphology, the smear of the sperm suspension was stained with Diff-Quick. A total of 200 cells were counted with the normal and abnormal morphology under the light microscopy 400×. Sperm viability was determined by eosin dye to estimate permeability of sperm membrane. Stained cells were considered as dead cells and no stained cells were considered as viable cells. A total of 200 sperm were evaluated.

**The effect of** ***Escanbil (Calligonum)*** **extract on apoptosis in the testes of aging groups**

TUNEL staining was used in order to estimate the number of apoptotic cells in testis tissue. In -Situ- Cell Death Detection Kit, POD from Roche company was used for this purpose. The paraffin-embedded testicular tissue were deparaffinized and rehydrate through a graded series of ethanols and double distilled water, washed in PBS, incubated for 15-30 min at 37^o^C with proteinase K, permeabilized with permeabilization solution (0.1% Triton x-100, 0.1% sodium citrate), incubated in TUNEL solution (450 μl of label solution added with 50μl of enzyme solution) at 37^o^C for 1 hour. Finally the samples were washed with PBS, they were observed under a fluorescent microscope (×400). Apoptotic cells were counted on a basement membrane of seminiferous tubules in each group. The total number of apoptotic cells was counted in 5 seminiferous tubules sections in each groups. We used thymus tissue as the positive control group. In negative control groups, sections incubated only with label solution instead of TUNEL reaction mixture. The data was analyzed by SPSS software(version 16) using ANOA test.

**Quantitative real-time polymerase chain reaction (RT-qPCR)**

Total RNA, was extracted according to the manufacturer's protocol kiasoul kit (Qigene) according to the protocol. In order to remove genomic contamination, RNA was treated with DNase I using a kit (Fermentase, Lithuania) based on the protocol described by the manufacturers. Concentrations of RNA were determined using UV spectrophotometer (Eppendorff, Germany). The cDNAs were synthesized from 500 ng DNase-treated RNA samples with a RevertAidTM first strand cDNA synthesis kit (Fermentase, Lithuania) using oligo (dT) primers. 

For PCR reactions, using Allele ID software, the desired primers, were designed (Catsper 1-4 and GAPDH gene as housekeeping and normalizer gene) (Table I). A total volume reaction was 20 μl, using 1000ng of cDNA, 1μl of forward primer,1 μl of reverse primer, 10 μl SYBR Green, 7μl RNAase free water that were added to each other. The real time-PCR cycling conditions were 95^o^C for 15 s, 60^o^C for 30 s, 72^o^C for 30 s, followed by 40 cycles. The temperature for melting curve program was determined between 60^o^C and 95^o^C. Efficiency was determined for each gene using a standard curve (the logarithmic dilution series of testis cDNA). We studied the expression of four genes from this family (Catsper1-4) in each group and each reaction was repeated three times. 

GAPDH gene as a reference gene was used. Expression levels of genes, according to formula pfaffel (the following method) were calculated. The data were analyzed by Spss using ANOVA test and the data evaluated as the mean±SD. The level of significance was assumed (p<0.05). In below it is described formula pfaffel that was used for qRT-PCR data analysis in the present study:

$\mathrm{Ratio}=\frac{\left(E_{t\arg\mathrm{et}}\right)∆Ct_{t\arg\mathrm{et}}}{\left(E_{\mathrm{reference}}\right)∆Ct_{\mathrm{reference}}}$

Ct_t arg et= _Ct_control_- Ct_treatment_

ΔCt_reference _= Ct_control _- Ct_treatment_

Ratio= 2^-ΔΔ^^Ct^ Equation 2

Whereas ΔΔCt= ΔCt_ reference _- ΔCt_t__arg__et_

**Statistical analyses**

Data were presented as mean±SD (standard deviation) and were analyzed using One-way repeated measure analysis of variance (ANOVA) followed by Tukey’s post hoc test. P-values<0.05 were considered statistically significant. 

## Results

Our results showed that after treatment with Calligonum extracts, sperm parameters in the group treated with doses of 30 mg/kg calligonum extract, improved compared to the other groups using other doses of this extract (p<0.05) (Table II). Also, the results of this study showed that after treatment with optimum dose (30 mg/kg) of *Escanbil (Calligonum) *extracts, sperm parameters in the each group changed based on table III.

**Ealuation of apoptosis in the testes of aging group after** ***Escanbil (Calligonum)*** **extract**

**Figure 1:**
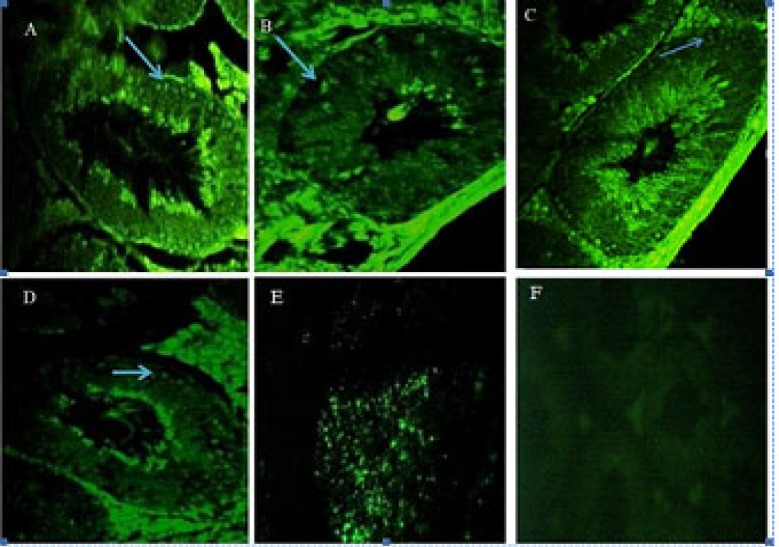
TUNEL staining indicated decrease of apoptosis of spermatogenic cells in 10 (A), 30 (B), 50 (C) mg/kg, aging control (D), Positive control (E) and Negative control (F). (The Original magnification ×400). The apoptosis rates of spermatogenic cells in (30mg/kg) testes were lower than those of 10 mg/kg and 50 mg/kg and aging control groups

**Figure 2:**
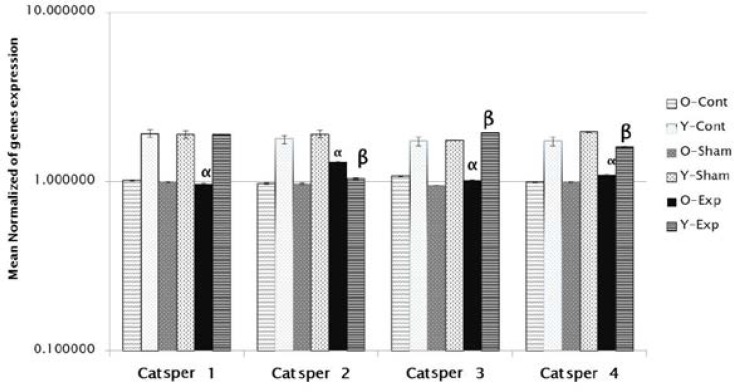
Mean normalized of gene expression indicated that the expression of Catsper 2, 4 was significantly enhanced in the aging experimental group compared with aging control group (p**<**0.05).

The results also indicate a decrease in apoptosis cells in the group treated with 30 mg/kg of *Escanbil (Calligonum) *extract compared to other groups (p<0.05) (Figure 1, Table IV). Our Data analysis showed a significant increase in gene expression Catsper 2, 4 in the aged experimental group compared to the aged control group (p<0.05). Gene expression in the younger group than the older group was significantly increased (p<0.05) (Figure 2).

**Table I:** Primers of catsper genes were designed for real time PCR reaction

**Gene**	**Primer sequences**	**Nucleotid (bp)**	**Tm (** ^o^ **C)**
Catsper1	F: GTTGTTGGACGACTCTCTGAC	21	83.7
	R: ACTTCTGTTGATGCTGTTCTACC	23	
Catsper2	F:CGAATGGGGCACATCACAC	19	83.1
	R:CGAGAAGACAGAACTATCAAGGAC	24	
Catsper3	F:ACTATCCTCTTCATCTTGCTTGC	23	82.8
	R:TTTGCTTCTCCTCCATAATCGC	22	
Catsper4	F:ACTATCCTCTTCATCTTGCTTGC	23	82.4
	R:TCGGTGCCTTCATTGGTCTC	20	
GAPDH	F:CGTTAATACCTCTTAATCCGGTT	21	84.3
	R:CGGTAACAACGGGCATTACT	20	

**Table II:** Evaluation of different doses of *Escanbil (Calligonum) *extracts on sperm parameters in aging male mice

** Sperm parameters**	Morphology (±SD)	Viability (±SD)	Total motility (±SD)	Count (SD± x×10^6^)
**Groups**				
**Control**	57.02 ± 1.16	58.68 ± 0.79	60.72 ± 1.41	4.11 ± 0.27
**Sham**	58.63 ± 0.92	58.63 ± 0.79	58.7 ± 2.22	4.3 ± 0.21
**10 mg/kg**	65.46 ±0.41^a^	58.16 ± 0.51	55.7 ± 1.02^a^^b^^d^	3.7 ± 0.78
**30 mg/kg**	68.32 ± 0.5^a^	69.38 ± 1.19 ab	69.78 ± 0.67 abc	4.7 ± 0.37 ce
**50 mg/kg**	65.70 ± 0.84^a^	57.90 ± 1.30	65.96 ± 0.7 ab	3.72 ± 0.75

a **:** **Statistical** **significance with control group (p** **<** **0.05)**

b **:** **Statistical significance with sham group (p** **<** **0.05)**

c **:** **Statistical significance with 10** **mg/kg group (p<0.05)**

d **:** **Statistical significance with 30** **mg/kg group (p<0.05)**

e **:** **Statistical significance with 50** **mg/kg group (p<0.05)**

**Table III:** The effect of optimum dose (30mg/kg) of *Escanbil (Calligonum) *extracts on sperm parameters of young and aging male mice

** Sperm parameters**	Morphology (±SD)	Viability (±SD)	Total motility (±SD)	Count (SD± x×10^6^)
**Groups**				
Aging control	57.02 ± 1.16	58.68 ± 0.79	60.72 ± 1.41	4.11 ± 0.27
Young control	68.54 ± 3.46 ab	72.58 ± 1.66 ab	74.3± 2.3^a^^b^	4.6 ± 0. 21 ab
Aging sham	58.63± 0.92	58.63 ± 0.79	58.7 ±2.22	4.3± 0.21
Young Sham	67.50 ± 0.17^a^^b^	71.46 ± 2.97^a^^b^	72.08 ± 3^a^^b^	4.7 ± 0.07 ab
Aging experiment	69.41 ±3.05 ab	68.34 ± 3.50^a^^b^	67.74 ± 2.14 ab	4.5 ± 0.56
Young experiment	76.94 ± 2.03 cde	58.82 ± 3.69^c^^d^^e^	73.30±1.02 e	4.90± 0.07

a **:** **Statistical significance with aging control group (p** **<** **0.05)**

b **:** **Statistical significance with aging sham group (p** **<** **0.05)**

c **:** **Statistical significance with young control group (p** **<** **0.05)**

d **:** **Statistical significance with young sham group** **(p<0.05)**

e **:** **Statistical significance with aging experiment group (p** **<** **0.05)**

**Table IV:** The effect of different doses of *Escanbil (Calligonum)* extract on the rate of apoptosis in spermatogonia cells in testis tissue

**Groups**	**Positive Tunel cells ± SD**
10 mg/kg	23±0.63
30 mg/kg	±14.2 2^a^^b^^c^
50 mg/kg	20±0.64 a
Aging control	25±2.5

a **: Statistical significance with aging control group (p** **<** **0.05)**

b **:Statistical significance with 10mg/kg group (p** **<** **0.05)**

c **:** **Statistical significance with 50mg/kg group (p< 0.05)**

α: Statistical significancy with aging control p**<**0.05

β: Statistical significancy with Young control group p**<**0.05.

O-cont: old (aging) control group

Y-cont: (young control group)

O-Sham: old (aging sham group)

Y-Sham: (young sham group)

O-Exp: aging experimental group

Y-Exp: young experimental group

## Discussion

The results of our study, showed improvement in sperm parameters such as viability, motility and morphology in the aged experiment group treated with 30 mg/kg *Escanbil (Calligonum) *extract compared to the aged control group. But antioxidant in this herbal extract had no effect on sperm count in aging experiment group comparison aging control group. Like our study scientists showed that antioxidants in pomegranate juice has a significant impact on the quality of sperm (5). Researchers have shown that antioxidants in the extract of Fumaria parviflora, causing an increase in the number of spermatogonia, spermatocyst, spermatozoa and Leydig cells (20). Using antioxidants of ginseng extract resulted in improving of sperm parameters like: sperm viability and sperm normal morphology in the experiment groups (21). Mohammadi *et al* and Hosseini *et al* have shown that treatment with selenium antioxidant causes an increase in sperm quality parameters such as: motility, viability and sperm normal morphology in the experiments groups (6, 21). The use of antioxidants in green tea extract resulted in improved sperm parameters in patients using the drug doxorubicin (22).

Our results also showed that only 30 mg/kg *Escanbil (Calligonum) *extract results in better survival of sperm while the higher and lower doses of this extract did not lead to the same conclusion. Like our study, scientists have shown that high doses of antioxidants, lead to lipid peroxidation and thus have a negative impact on sperm parameters (23). The use of high-dose antioxidants albumin leads to lipid peroxidation (2). In the present study, administration of *Escanbil (Calligonum) *extract enhanced normal morphology cells some of the researchers like Aziz *et al* have shown an association between ROS and the formation of spermatozoa with abnormal morphology (2). These findings demonstrate our results. Scientists believe that increase in ROS leads to the loss of epithelial cells which in turn leads to damage of the Sertoli cells and then cytoplasmic bridges collapsed and eventually can cause abnormal sperm (24).

In our study apoptosis in the testes of aged control group was observed. Researchers have found that aging causes decreased sperm motility and increased DNA fragmentation in spermatozoa. Findings of researchers have shown that ROS, cause DNA fragmentation and thus increase infertility (3). In this study, the Catsper gene expression levels before and after administration of Escanbil* (Calligonum) *extract were evaluated in aged and young groups. Antioxidants present in the *Escanbil (Calligonum) *extract could alter Catsper gene expression. Scientists have shown that Quercetin (an important antioxidant in calligonum extract) can alter gene expression of TNFα in lupus patients (25).

Kaempferol is another antioxidant which is present in *Calligonum* extract. Kaempferol can cause changes in gene expression (26). In our present study analyzed data indicates that the Catsper gene expression decreased in the aged group compared to the younger group. Then aging can cause decreasing of gene expression. Cao et al have shown that aging causes a decrease in the expression of antioxidant genes (27). In the present study, the Catsper gene expression increased in young and elderly groups after administration of the Calligonum extract. Evidence for sperm parameters analyzes in these groups, confirms these findings. Although the administration of this extract led to increased expression of Catsper 2,4 but it also led to reduced Catsper 1,3 gene expression in aged experiments group compared to the aged control group Indicating that this extract has a greater impact on expression of Catsper 2,4 than other genes in this family. 

In the present study the Catsper gene expressed in 2-3 months young groups Nikpoor *et al* showed that the Catsper gene expresses as early as 3 weeks of age (28). The expression of Catsper gene in young group showed the up regulation of Catsper 3, 4, indicating that *Escanbil (Calligonum) *extract has more effects on Catsper 3, 4 subunits in young group. We observed down regulation of Catsper 2, in young groups after *Calligonum* treatment too. We found that antioxidant components in *Escanbil (Calligonum) *extract (30 mg/kg) can alter Catsper gene expression in young and aged groups; however these changes don’t follow the same pattern.

The Sperm parameters such as motility and morphology are affected by ROS (29). Nowadays, scientists have shown that some plants have important antioxidant components (30). *Calligonum* comusum is an example of a medicinal plant. Scientists have shown that *Calligonum* comusum has the important antioxidants such as: Catechin, Kaempferol, Quercetin and Epicatechin (8). Badria *et al* showed that *Calligonum* camosum has two other antioxidants such as Genistin and Isoprunetin (8). Liu *et al* showed that *Calligonum* extract has anti-inflammatory properties (31). Here, we chose *Calligonum* Como sum for our study because of these features. Scientists believe that antioxidants have a significant impact on mitochondria energetic metabolism. As in the present study, Garolla showed that the use of antioxidants can lead to significant improvement in sperm motility (32).while another study showed that Aloe vera gel Extract has negative effect on sparm motility (33).

In the present study, sperm survival rate was estimated using the cell permeable dye (Eosin). The *Escanbil (Calligonum) *extract (30 mg/kg) led to a decrease in sperm membrane permeability, resulting in increased cell survival in experiments groups. Kobayashi *et al* have proved that increasing the survival rate of sperm cells is associated with reduced levels of ROS (34). We used aging mice (11-13 months) in our study and found that sperm parameters in aging groups will be decreased compared to young group. Scientists believe that sperm parameters decrease with aging. Some scientists believe that aging has a detrimental effect on sperm motility, while other scholars reject this thread (3). In the aging subject atrophy in smooth muscles in prostate and decreasing in its protein content may affect sperm count and sperm motility (35).

In our study we showed that, antioxidants can change the expression of gene. A study showed that Catechin, Quercetin and flavonoid can change the glutathione peroxidase gene expression in experiments groups (10) In our study, antioxidant in *Escanbil (Calligonum) *extract 30 mg/kg could change expression of catsper gene. Like our study, Kanakis *et al* and Mohammadi *et al* showed that using of antioxidants like selenium; L carnitine can lead to up regulation of Catsper 1 gene (25, 35-37). 

## Conclusion

Our data showed that the antioxidant in Calligonum extract (30 mg/kg) can improve sperm parameters and alter the Catsper genes expression in aged male mice. So, this herbal extract can be used as an antioxidant component for clinical usages.
